# Supplementary material for: The Baraitser–Winter Cerebrofrontofacial Syndrome Recurrent R196H Variant in Cytoplasmic β‐Actin Impairs Its Cellular Polymerization and Stability
Source: FASEB J. 2026 Jan 5;40(1):e71386. doi: 10.1096/fj.202502196R (PMC12767641; doi:10.1096/fj.202502196R)
Supplement: Supplementary file 1 — Figure S1. Supplementary to Figure 2 R196H mutation reduces proliferation and migration of patient‐derived fibroblasts cells. Figure S2. Supplementary to Figure 4 R196H mutation impairs actin filament organization in patient‐derived fibroblasts. Figure S3. Supplementary to Figure 6 R196H mutation affects the attachment of actin to the plasma membrane. Figure S4. Supplementary to Figure 7 R196H mutation increases the dynamic reorganization of actin by enhancing cofilin dissociation. Table S1. Mean values ± SD of apparent elastic modulus (E i) and equilibrium elastic modulus (E r), F peak, F e, tether length (L), tether radius (r t), patch radius (R p), and normalized peak force (F*peak) for all experimental conditions. [file FSB2-40-e71386-s001.docx]

**Supplementary material**

**The Baraitser-Winter Cerebrofrontofacial Syndrome recurrent R196H variant in cytoplasmic β-actin impairs its cellular polymerization and stability**

Éva Gráczer^1^, Elena Battirossi^2^, Tamás Bozó^1^, Áron Gellért Altorjay^1^, Katalin Pászty^1^, Laura Harsányi^1^, Johannes N. Greve^3^, Irene Pertici^2^, Massimo Reconditi^2^, Nataliya Di Donato^4^, Miklós Kellermayer^1^, Pasquale Bianco^2,*^ and Andrea Varga^1,*^

^1^Department of Biophysics and Radiation Biology, Semmelweis University, Budapest, Hungary

^2^PhysioLab, University of Florence, Firenze, Italy

^3^Institute for Biophysical Chemistry, Hannover Medical School, 30625 Hannover, Germany

^4^Department of Human Genetics, Hannover Medical School, 30625 Hannover, Germany

*Correspondence: matkovicsne.andrea@semmelweis.hu

pasquale.bianco@unifi.it

**Figure S1**, supplementary to Fig. 2 **R196H mutation reduces proliferation and migration of patient-derived fibroblasts cells**

**Figure S2**, supplementary to Fig. 4 **R196H mutation impairs actin filament organization in patient-derived fibroblasts**

**Figure S3**, supplementary to Fig. 6 **R196H mutation affects the attachment of actin to the plasma membrane**

**Figure S4**, supplementary to Fig. 7 **R196H mutation increases the dynamic reorganization of actin by enhancing cofilin dissociation**

**Table S1 Mean values ± SD of apparent elastic modulus (*E*_i_) and equilibrium elastic modulus (*E*_r_), *F*_peak_, *F*_e_, tether length (*L*), tether radius (*r*_t_), patch radius (*R*_p_), and normalized peak force (*F*^*^_peak_) for all experimental conditions.**

**Figure S1**, supplementary to Fig. 2 **R196H mutation reduces proliferation and migration of patient-derived fibroblasts cells**

**(A)** Confocal images of wild type and R196H mutant fibroblast monolayers stained for actin (phalloidin staining, gray), ZO-1 (gray), pFAK (yellow) and cell nuclei (Hoechst staining, blue). The average size **(B)** and the number **(C)** of pFAK spots in each field of view (120 × 120 μm area) were determined and illustrated for wild type and R196H mutant fibroblasts. Data were analyzed by one-way ANOVA, followed by Bonferroni’s multiple comparison test expressed as mean ± SD (*p < 0.05).

**
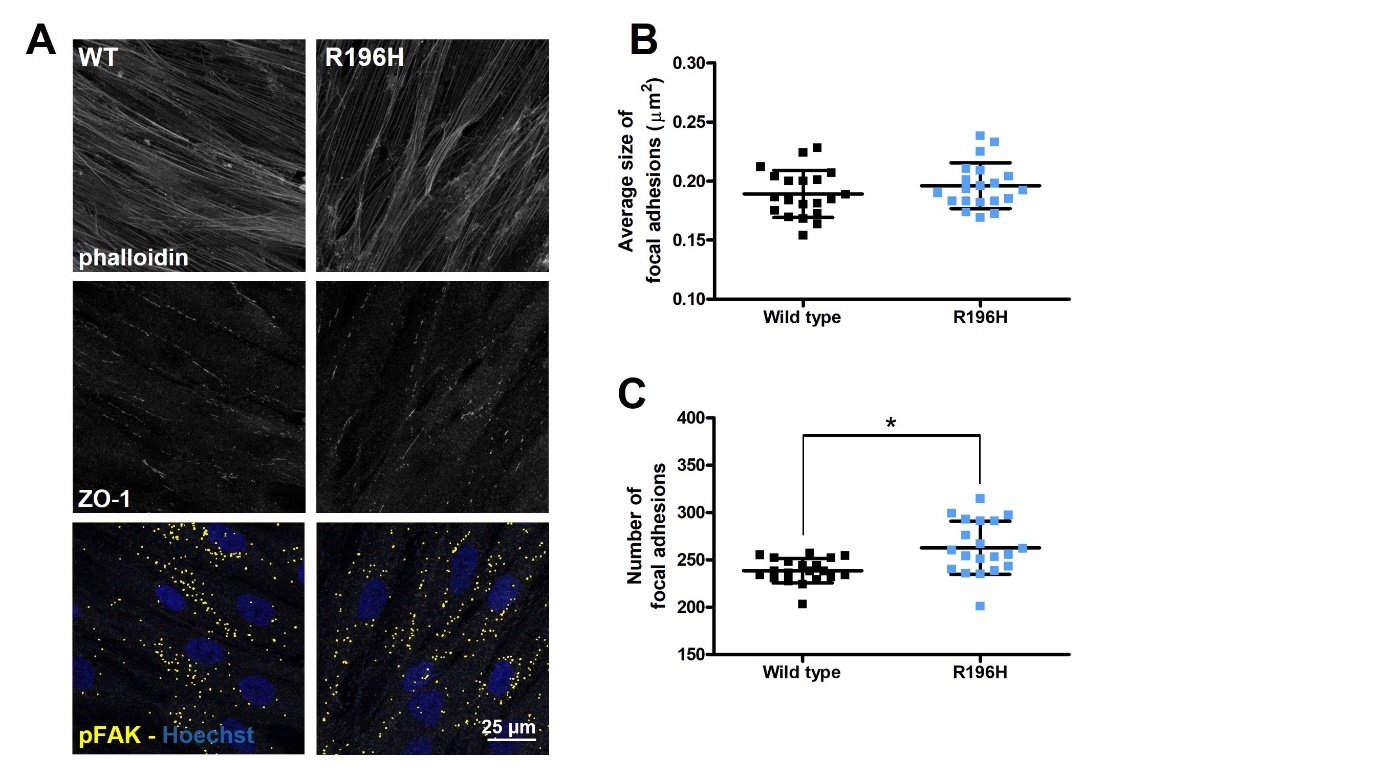
**

**Figure S2**, supplementary to Fig. 4 **R196H mutation impairs actin filament organization in patient-derived fibroblasts**

Pairwise comparison of the histograms of actin width determined for wild type and the R196H fibroblasts **(A)**, or to compare the effects of jasplakinolide either on wild type **(B)** or on the R196H fibroblasts **(C)**. Examples of directionality histograms created by ImageJ, 1.53c for the pairs of wild type **(D)** and the R196H **(E)** fibroblasts related to Figure 4C-E, as well as for the pairs of wild type fibroblasts treated with either DMSO **(F)** or CK-666 **(G)** related to Figure 4F and G.

**
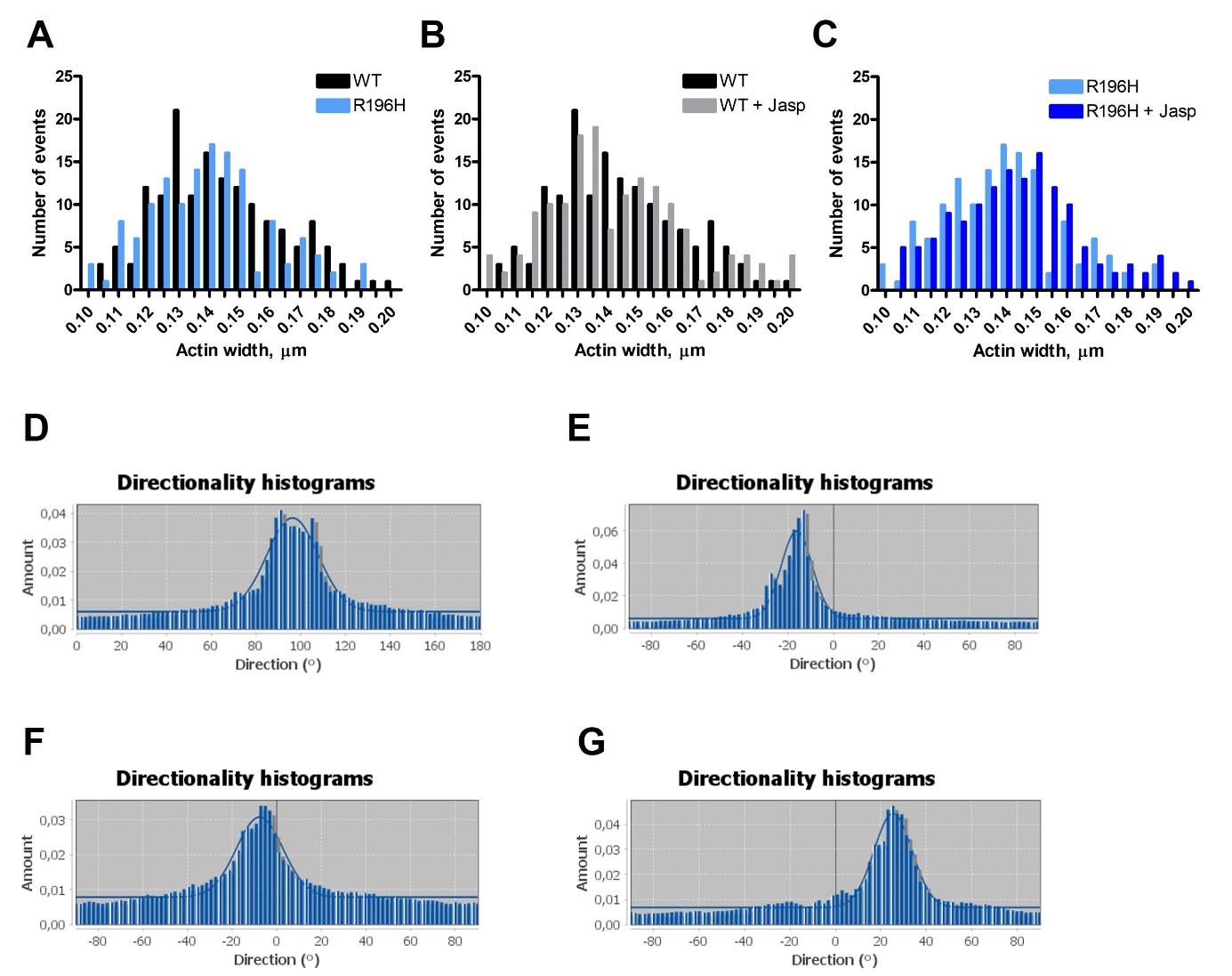
**

**Figure S3**, supplementary to Fig. 6 **R196H mutation affects the attachment of actin to the plasma membrane**

**(A)** Cell membrane indentation with a ramp-shaped push (*v* = 4 μm/s). The indentation begins when the membrane's position (*L*, black) deviates from the nanopositioner (*X*, red), indicating the development of resisting force (*F*, blue). Force increases linearly during the ramp, followed by a relaxation phase, revealing the viscoelastic nature of the response. The relaxation follows a double-exponential decay and reaches near equilibrium at *F*_r_. Inset shows vertically zoomed *L* and *X* traces within the green box. *d*_i_ and *d*_r_ show the indentation depth at the end of the push and after the relaxation, respectively. Orange and yellow dashed lines: double-exponential fit on the traces. The magenta vertical dashed line marks the start of the indentation, the black vertical dashed lines indicate the ramp ends. **(B)** Schematic representation of a five-element Zener model used for identification of the mechanical parameters explaining the response of the system. The model in its simplest version (Maxwell model) comprises two parallel branches: one with a spring (*k_0_*) representing the purely elastic component (static), and the other with a series combination of a spring (*k_1_*) and dashpots (𝛾*_1_*) representing the viscoelasticity responsible for the relaxation process. The biphasic appearance of the relaxation can be accounted for by adding in parallel a second series combination of a spring (*k_2_*) and a dashpot (𝛾*_2_*) (Zener model). **(C)** Tether size measurements using the DLOT imaging. The figure shows a representative image of a tether extracted from wild type fibroblasts. Inset illustrates the transversal intensity profiles of the tether measured within the yellow dashed rectangles. To compare the tether radius among different experimental models, avoiding possible changes associated with tether length, all measurements were conducted on tethers with a narrow range (38–43 μm) of lengths.

**
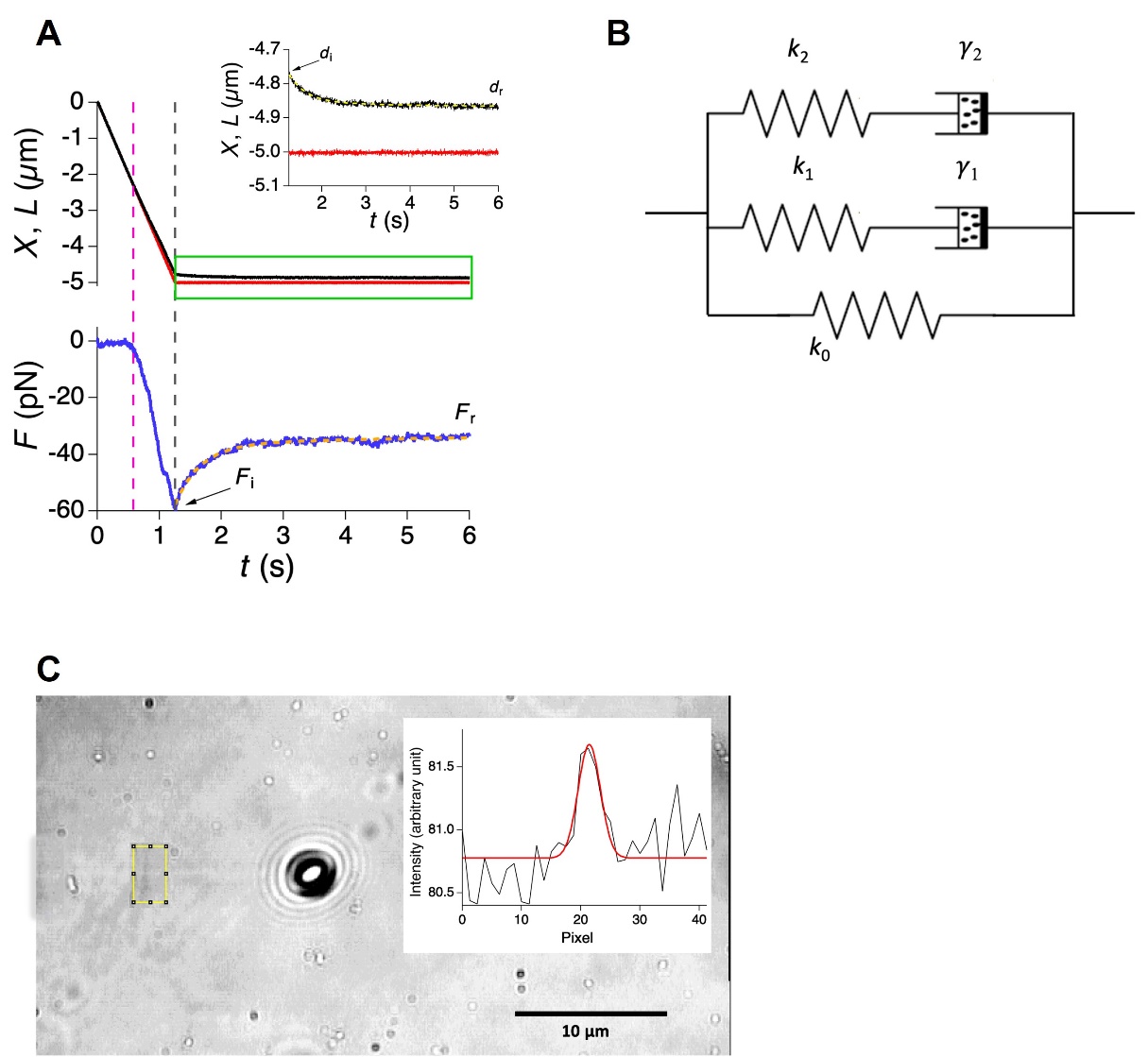
**

**Figure S4**, supplementary to Fig. 7 **R196H mutation increases the dynamic reorganization of actin by enhancing cofilin dissociation**

**(A-H)** Immunofluorescence analysis of wild type (A, B, E, F) or R196H (C, D, G, H) fibroblasts kept either non-stretched (A, C, E, G) or stretched (B, F, D, H) for 15 minutes. Samples were stained with either phalloidin (A-D) or for cofilin (E-H). Examples of areas detected in non-stretched (blue) or stretched (green) confocal images for visual comparison are shown.

**
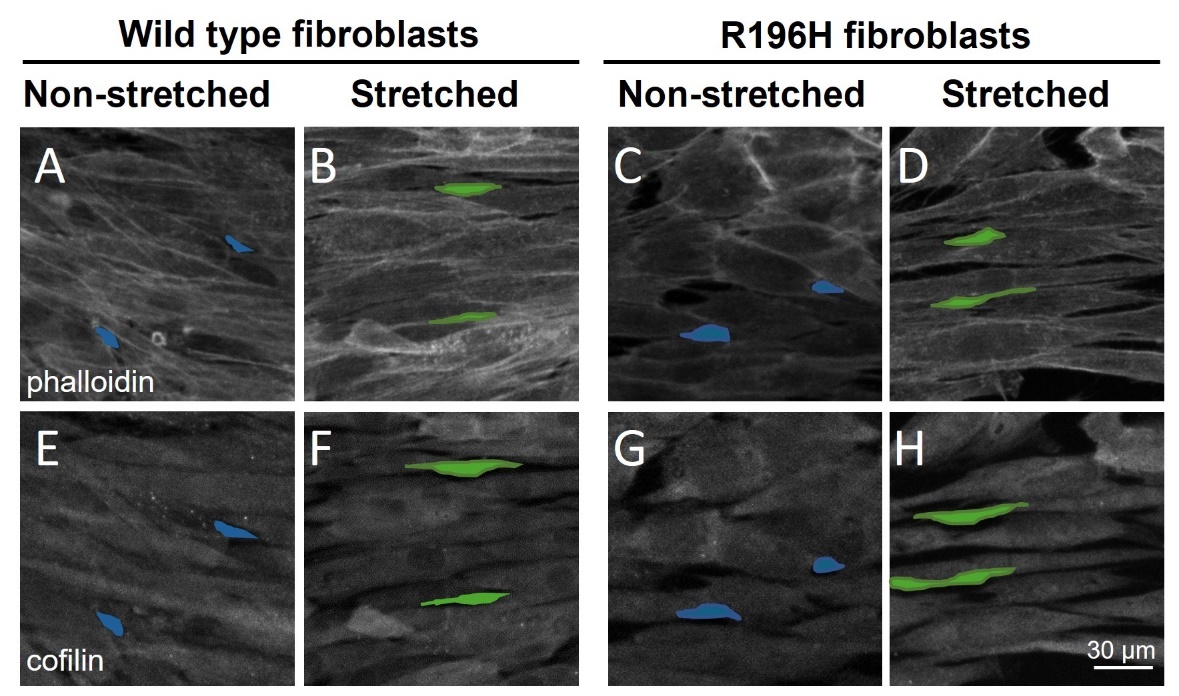
**

**Table S1. Mean values ± SD of apparent elastic modulus (*E*_i_) and equilibrium elastic modulus (*E*_r_), *F*_peak_, *F*_e_, tether length (*L*), tether radius (*r*_t_), patch radius (*R*_p_), and normalized peak force (*F*^*^_peak_) for all experimental conditions.** Measurements were performed on wild type fibroblasts (WT), fibroblasts expressing the R196H mutation, and wild type fibroblasts treated with 5 μM Latrunculin A (Latr). *R*_p_ was calculated based on the tether radius (*r*_t_) measured using the DLOT imaging setup. *F*_peak_ and *F*_e_ values were analyzed at tether lengths ranging from 38 to 43 μm, with a retraction speed of 4 µm/s. *n* indicates the number of cells analyzed under each specific protocol: push or pull. Statistical significance vs. WT: # *p* < 0.05; ## *p* < 0.01; ### *p* < 0.001.

|  | *E*_i_ (Pa) | *E*_r_ (Pa) | *F*_peak_ (pN) | *F*_e_ (pN) | *L* (µm) | *r*_t_ (nm) | *R*_p_ (nm) | *F*^*^_peak_ (pN/nm^2^) |
| --- | --- | --- | --- | --- | --- | --- | --- | --- |
| WT | 57.7 ± 19.8 (*n*=31) | 21.9 ± 7.9 | 138 ± 25  (*n*= 5) | 25 ± 4 | 42 ± 1 | 108 ± 6 | 949 ± 122 | 49 ± 7 |
| R196H | 71.3 ± 28.3 (*n*=26) | 21.3 ± 7.4 | 83 ± 20^#^  (*n*= 5) | 17 ± 2 | 42 ± 1 | 117 ± 12 | 910 ± 111 | 32 ± 6^#^ |
| Latr | 10.2 ± 5.1 ^#^ (*n*=19) | 5.3 ± 2.1 ^#^ | 51 ± 12^##^  (*n*= 5) | 4 ± 1 | 39 ± 4 | 184 ± 6^##^ | 4324 ± 1048^##^ | 0.87 ± 0.20^###^ |
